# Melatonin against acute ischaemic stroke dependently via suppressing both inflammatory and oxidative stress downstream signallings

**DOI:** 10.1111/jcmm.15654

**Published:** 2020-07-30

**Authors:** Kuan‐Hung Chen, Kun‐Chen Lin, Sheung‐Fat Ko, John Y. Chiang, Jun Guo, Hon‐Kan Yip

**Affiliations:** ^1^ Department of Anesthesiology Kaohsiung Chang Gung Memorial Hospital and Chang Gung University College of Medicine Kaohsiung Taiwan; ^2^ Department of Radiology Kaohsiung Chang Gung Memorial Hospital and Chang Gung University College of Medicine Kaohsiung Taiwan; ^3^ Department of Computer Science and Engineering National Sun Yat‐Sen University Kaohsiung Taiwan; ^4^ Department of Cardiology The First Affiliated Hospital Jinan University Guangzhou China; ^5^ Division of Cardiology Department of Internal Medicine Kaohsiung Chang Gung Memorial Hospital and Chang Gung University College of Medicine Kaohsiung Taiwan; ^6^ Institute for Translational Research in Biomedicine Kaohsiung Chang Gung Memorial Hospital Kaohsiung Taiwan; ^7^ Center for Shockwave Medicine and Tissue Engineering Kaohsiung Chang Gung Memorial Hospital Kaohsiung Taiwan; ^8^ Department of Medical Research China Medical University Hospital China Medical University Taichung Taiwan; ^9^ Department of Nursing Asia University Taichung Taiwan; ^10^ Division of Cardiology Department of Internal Medicine Xiamen Chang Gung Hospital Fujian China

**Keywords:** inflammation, ischaemic stroke, melatonin, oxidative stress, Toll‐like receptors

## Abstract

This study tested the hypothesis that melatonin (Mel) therapy preserved the brain architectural and functional integrity against ischaemic stroke (IS) dependently through suppressing the inflammatory/oxidative stress downstream signalling pathways. Adult male B6 (n = 6 per each B6 group) and TLR4 knockout (ie TLR4^−/−^) (n = 6 per each TLR4^−/−^ group) mice were categorized into sham control (SC^B6^), SC^TLR4−/−^, IS^B6^, IS^TLR4−/−^, IS^B6^ + Mel (i.p. daily administration) and IS^TLR4−/−^ + Mel (i.p. daily administration). By day 28 after IS, the protein expressions of inflammatory (HMBG1/TLR2/TLR4/MAL/MyD88/RAM TRIF/TRAF6/IKK‐α/p‐NF‐κB/nuclear‐NF‐κB/nuclear‐IRF‐3&7/IL‐1β/IL‐6/TNF‐α/IFN‐γ) and oxidative stress (NOX‐1/NOX‐2/ASK1/p‐MKK4&7/p‐JNK/p‐c‐JUN) downstream pathways as well as mitochondrial‐damaged markers (cytosolic cytochrome C/cyclophilin D/SRP1/autophagy) were highest in group IS^B6^, lowest in groups SC^B6^ and SC^TLR4−/−^, lower in group IS^TLR4−/−^ + Mel than in groups IS^TLR4−/−^ and IS^B6^ + Mel and lower in group IS^B6^ + Mel than in group IS^TLR4−/−^ (all *P* < .0001). The brain infarct volume, brain infarct area and the number of inflammatory cells in brain (CD14/F4‐88) and in circulation (MPO+//Ly6C+/CD11b+//Ly6G+/CD11b+) exhibited an identical pattern, whereas the neurological function displayed an opposite pattern of inflammatory protein expression among the six groups (all *P* < .0001). In conclusion, TLR inflammatory and oxidative stress signallings played crucial roles for brain damage and impaired neurological function after IS that were significantly reversed by Mel therapy.

## 1 INTRODUCTION

Previous studies^1^, ^2^, ^3^, ^4^, ^5^ have demonstrated that acute ischaemic stroke (IS) elicits a vigorous inflammatory reaction, augments the generation of cytokines and oxidative stress and initiates the complement cascade. These factors, in turn, further aggravate brain damage after acute IS,^1^, ^2^, ^3^, ^4^, ^5^, ^6^, ^7^ causing irreversibly neurological sequelae.^1^, ^2^, ^3^, ^4^, ^5^, ^6^, ^7^ Of these inflammatory mediators, damage‐associated molecular patterns (DAMPs) have been identified as a critical signal that triggers the inflammatory immune system in response to tissue damage.^8^, ^9^, ^10^ DAMPs initiate signalling cascades that activate Toll‐like receptors (TLRs) and myeloid differentiation factor 88 (MyD88), and further activate the downstream signalling of IκB, nuclear factor (NF)‐κB, interleukin (IL)‐β, tumour necrosis factor (TNF) and interferon (IFN) regulatory factors, etc.^8^, ^9^, ^10^, ^11^ Some investigators have further reported that expressions of human TLR4 in plasma were associated with poor outcome and stronger inflammatory response in acute IS.^12^ Melatonin (Mel) and its derivatives are characterized with potent‐free radical scavengers^13^, ^14^ and have been displayed to play a key role in retaining cell membrane stability and ensuring cell survival in a toxic environment mainly through reduction in susceptibility to oxidative stress and free radical damage as well as suppression of inflammatory reaction.^15^, ^16^, ^17^, ^18^, ^19^

Intriguingly, abundant experimental studies have previously shown that Mel treatment effectively reduced brain infarct volume and preserved neurological function and the integrity of brain architecture in different species of animals after acute IS.^20^, ^21^, ^22^, ^23^ Surprisingly, although the benefit of Mel on protecting the brain against the IS damage has been extensively investigated in animal model of acute IS, there is a lack in any clinical study to investigate whether Mel treatment would be safe and offers additional benefit for the patients after IS. This issue raises the fundamental requirement to more completely understand the mechanism for why the Mel would alleviate the brain infract volume and preserve the neurological function in setting of IS prior to conducting such a clinical trial. Interestingly, some investigators have revealed that Mel attenuated TLR4‐mediated inflammatory response through MyD88‐ and Toll‐like receptor‐associated activator of interferon (TRIF)‐dependent signalling pathways in animal model of ovarian cancer^24^ as well as Mel protected the myocardium against brain death tissue extract damage mainly through suppressing the DAMP‐TLR2/TLR4‐mediated MyD88‐NF‐κB inflammatory downstream signalling.^10^ Accordingly, this study tested whether Mel treatment improved neurological outcome in mouse IS through inhibiting DAMP‐TLR‐MyD88/TRIF‐mediated inflammatory and oxidative stress downstream signallings.

## 2 MATERIALS AND METHODS

### 2.1 Ethics statement

All animal experimental procedures were approved by the Institutional Animal Care and Use Committee at Kaohsiung Chang Gung Memorial Hospital (Affidavit of Approval of Animal Use Protocol No. 2017082202) and performed in accordance with the Guide for the Care and Use of Laboratory Animals, 8th edition.

### 2.2 Animal grouping and rationale of mel dosage and time‐points of treatment

Pathogen‐free, 12‐week‐old male C57B/L6 (ie B6) mice (n = 18, ie 6 animals per each B6 subgroup) and TLR^4−/−^mice (n = 18, ie 6 animals per each TLR^4−/−^ subgroup) were equally categorized into six groups: groups 1 [sham‐operated control (SC^B6mouse^)], 2 (SC^TLR4−/−mouse^), 3 (IS^B6mouse^), 4 (IS^TLR4−/−mouse^), 5 [IS^B6mouse^ + Mel (50 mg/kg 3 days prior to and 3 days after acute IS procedure, followed by 20 mg/kg/d by up to 28 days by peritoneal injection)] and 6 (IS^TLR4−/−mouse^ + Mel with the same dosage and time‐points of treatment as group 5). The dosage and time‐points of treatment of Mel were based on our previous reports^10^, ^17^, ^18^, ^19^, ^24^ with minimal modification. Melatonin, which is safe and non‐toxic in human beings, is popularly utilized as a dietary supplement. Previous randomized control trial had demonstrated that intravenous administration of 50 mg Mel was safe for patients with acute myocardial infarction.^25^ Based on the above‐mentioned information, an extrapolation to the human dosage of 5 to 10 mg Mel for daily should be very safe.

### 2.3 The genetic background of TLR4^−/−^ mice

TLR4^−/−^ mice (B6.B10Scn‐Tlr4lps‐del/JthJ) used in the present study were purchased from Jackson Laboratory. Although the original genetic background of TLR4^−/−^ mice was C57BL/10Scn and crossed to B6 (C57BL/6), the utilization of C57BL/6 mice as the wild‐type control for TLR4^−/−^ mice was also found in other recent study.^26^, ^27^ Hence, we suggest that the genetic background of mice should not affect the results and conclusion of the present study.

### 2.4 Cell culture

N2a cells were maintained in MEM supplemented with 10% foetal bovine serum, 2 mmol/L L‐glutamine, 0.1 mmol/L non‐essential amino acids, 0.1 mmol/L sodium pyruvate, 100 U/mL penicillin G and 100 µg/mL streptomycin.

### 2.5 shRNA TLR2/TLR4 double knockdown N2a cells for assessment of TLR‐dependent inflammatory downstream signallings

shRNA clones were obtained from the National RNAi Core Facility Platform located at the Institute of Molecular Biology‐Genomic Research Center, Academia Sinica. Individual clones were identified by their unique TRC number (shTLR4:TRCN0000065786, shTLR2:TRCN0000321317, shLacZ:TRCN0000072233). Transient transfection of cells with plasmids was performed with Lipofectamine 3000 according to the manufacturer's instructions but with slight modifications. Following the IS^B6‐mouse^ brain tissue extract (BE) (240 μg/mL) or Mel (100 μmol/L) treatment, transfected cells were lysed with RIPA buffer and performed with SDS‐PAGE analysis.

### 2.6 Procedure of acute is, and advantage and disadvantage of this model

The procedure for the experimental model of acute IS has been described in our previous studies^28^, ^29^ with some modifications. Each animal was anaesthetized by 2% inhalational isoflurane. After exposure of the left common carotid artery (LCCA) through a transverse neck incision, a small arteriotomy was performed on the LCCA through which silicon rubber‐coated monofilament (ie 0.22 ± 0.01 mm diameter) was carefully advanced into the distal left internal carotid artery for occlusion of the left middle cerebral artery, causing brain ischaemia and infarction of its supplied area. The nylon monofilament was removed 50 minutes after occlusion, followed by closure of the muscle and skin in layers. For groups 1/2 (sham‐operated control), only neck skin and muscle layers were opened, followed by closing these two layers. The advantage of this animal model is reproducible and could provide consistently neurological dysfunction for the study. On the other hand, the disadvantage of this animal model is quite difficult for a new investigator to operate.

### 2.7 Corner test for assessment of neurological function prior to and after is induction

The sensorimotor functional test (corner test) was conducted for each rat of each group (ie n = 6 per group) at baseline and on days 1, 3, 7, 14 and 28 after acute IS induction as we previously described.^28^, ^29^ In detail, the mice could walk through a tunnel and then turn into a 60‐degree corner. To exit the corner, the mice could turn either left or right. The results were recorded by a technician blinded to the study design. This test was repeated 10 to 15 times with at least 30 seconds between each trial. We recorded the number of right and left turns from 10 successful trials for each animal and used the results for statistical analysis.

### 2.8 Procedure and protocol of brain magnetic resonance imaging (MRI) for determining the brain infarct volume (BIV)

The procedure and protocol for brain magnetic resonance imaging (MRI) study were based on our previous report.^29^ The MRI was performed at day 28 after IS induction. Briefly, during MRI measurements, mice were anaesthetized by 2% inhalational isoflurane with room air and placed in an MRI‐compatible holder (Biospec 94/20). Rectal temperature and respiration were monitored throughout the procedure to ensure normal physiological conditions were maintained. MRI data were collected using a Varian 9.4T animal scanner (Biospec 94/20) with a rat surface array. The MRI protocol consisted of 40 T2‐weighted images. Forty continuous slice locations were imaged with a field of view of 30 mm × 30 mm, an acquisition matrix dimension of 256 × 256 and slice thickness of 0.5 mm. The repetition time (TR) and echo time (TE) for each fast spin echo volume were 4200 ms and 30 ms, respectively. Custom software, ImageJ (1.43i, NIH), was used to process the region of interest (ROI). Planimetric measurements of images from MRI T2 were performed to calculate the stroke volumes of cortex. Collectively, the BIV was calculated by summation of total coronal sections and then divided the numbers of coronal sections to obtain the means of infarct areas. Additionally, the height of the infarct zone was calculated by summation of the thickness of each coronal section. Finally, the BIV was obtained by mean of infarct area × height.

### 2.9 Identification of brain infarct area (BIA) at day 14 after acute is procedure

For 3,5‐triphenyl‐2H‐tetrazolium chloride (TTC) (Alfa Aesar) stain, additional four animals in each group were utilized in the present study. The procedure and protocol have been described in our previous reports.^29^, ^30^ The sections were photographed directly above at a fixed height. The images obtained were then analysed using ImageTool 3 (IT3) image analysis software (University of Texas, Health Science Center, San Antonio, UTHSCSA; Image Tool for Windows, version 3.0).

### 2.10 Immunofluorescent (IF) staining

The procedure for IF staining has been described in our previous reports.^29^, ^30^ For IF staining, rehydrated paraffin sections were first treated with 3% H_2_O_2_ for 30 minutes and incubated with Immuno‐Block reagent (BioSB) for 30 minutes at room temperature. Sections were then incubated with primary antibodies specifically against CD14 (1:100, Santa Cruz) and F4/80 (1:100, Santa Cruz), whereas sections incubated with the use of irrelevant antibodies served as controls. Three sections of brain specimen from each animal were analysed. For quantification, three random HPFs (400× for IF studies) were analysed in each section. The mean number of positively stained cells per HPF for each animal was then determined by summation of all numbers divided by 9.

### 2.11 Western blot analysis

The procedure for Western blot analysis was based on our recent reports.^28^, ^29^, ^30^ Briefly, equal amounts (50 μg) of protein extracts were loaded and separated by SDS‐PAGE using acrylamide gradients. After electrophoresis, the separated proteins were transferred electrophoretically to a polyvinylidene difluoride membrane (GE, UK). Non‐specific sites were blocked by incubation of the membrane in blocking buffer [5% non‐fat dry milk in T‐TBS (TBS containing 0.05% Tween‐20)] overnight. The membranes were incubated with the indicated primary antibodies [high mobility group box 1 (HMGB1) (1:1000, Cell Signaling), Toll‐like receptor (TLR)2 (1:1000, Abcam), TLR4 (1:1000, Novus), tumour necrosis factor (TNF) receptor‐associated factor 6 (TRAF6) (1:2000, Abcam), myeloid differentiation primary response 88 (MyD88) (1:1000, Abcam), nuclear factor of kappa light polypeptide gene enhancer in B‐cell inhibitor kinase (IKB)‐α (1:1000, Cell Signaling), phosphorylated (p)‐IκB‐α (1:1000, Cell Signaling), inhibitor of nuclear factor kappa‐B kinase subunit alpha (IKK‐α) (1:5000, Abcam), MyD88 adaptor‐like (MAL) (1:1000, Abcam), TIR domain‐containing adapter‐inducing interferon‐β (TRIF) (1:1000, Abcam), translocation‐associated membrane protein (TRAM) (1:1000, Thermo Fisher Scientific), interferon regulatory factor 1 (IRF3) (1:1000, Cell Signaling), phosphorylated (p)‐IRF3 (1:1000, Cell Signaling), IRF7 (1:5000, Thermo Fisher Scientific), p‐IRF7 (1:1000, Thermo Fisher Scientific), nuclear factor (NF)‐κB (1:1000, Abcam), phosphorylated (p)‐NF‐κB (1:1000, Cell Signaling), tumour necrosis factor (TNF)‐α (1:1000, Cell Signaling), interleukin (IL)‐1β (1:1000, Cell Signaling), IL‐6 (1:1000, Biorbyt), induced nitric oxide synthase (iNOS) (1:1000, Abcam), interferon (IFN)‐γ (1:5000, Abcam), phosphorylated mitogen‐activated protein kinase kinase 4 (p‐MMK4) (1:1000, Cell Signaling), p‐MMK7 (1:1000, Thermo Fisher Scientific), phosphorylated c‐Jun N‐terminal kinase (p‐JNK) (1:1000, Abcam), p‐c‐JUN (1:1000, Abcam), cytosolic cytochrome C (1:1000, BD), cyclophilin D (1:1000, Abcam), dynamin‐related protein 1 (DRP1) (1:1000, Abcam), LC3B‐II (1:2000, Abcam), LC3B‐I (1:2000, Abcam), mitochondrial Bax (1:1000, Abcam), caspase 3 (1:1000, Cell Signaling), caspase 9 (1:1000, Cell Signaling), NOX‐1 (1:1500, Sigma), NOX‐2 (1:750, Sigma), apoptosis signal‐regulating kinase 1 (ASK1) (1:1000, Abcam) and actin (1:1000, Millipore)] for 1 hour at room temperature. Horseradish peroxidase‐conjugated anti‐rabbit immunoglobulin IgG (1:2000, Cell Signaling) was used as a secondary antibody for one‐hour incubation at room temperature. The washing procedure was repeated eight times within one hour. Immunoreactive bands were visualized by enhanced chemiluminescence (ECL; Amersham Biosciences) and exposed to BioMax L film (Kodak). For the purpose of quantification, ECL signals were digitized using Labwork software (UVP).

### 2.12 Flow cytometric quantifications of circulatory inflammatory cells

The peripheral blood was sampled in each group at day 3 after acute IS induction. The numbers of inflammatory cells were firstly doubly stained with specific antibodies for MPO+, Ly6C+/CD11b+ and Ly6G+/CD11b+ cells, respectively. The inflammatory cells were measured at 72 hours due to the peak level of circulatory inflammatory biomarkers (ie including those of inflammatory cells and proinflammatory cytokines) frequently occurring at the time intervals of 12‐72 hours after an acute inflammatory reaction.

### 2.13 In vitro pilot study for elucidating the TLR‐dependent inflammatory downstream signalling protein expressions (refer to Figure 1)

To clarify the inflammatory signalling pathway in setting of acute IS, N2a cells with and without TLR2/TLR4 double knockdown were utilized in this pilot study and were categorized into the four groups: wild‐type (N2a cells only), TLR2/TLR4 double knockdown (TLR^2‐/4‐^) N2a cells, Na2 cells or TLR^2‐/4‐^ N2a cells with and without IS^B6‐mouse^ brain tissue extract treatment, and N2a cells or TLR^2‐/4‐^ N2a cells with and without Mel treatment.

For obtaining IS^B6‐mouse^ brain tissue extract, additional four IS B6 mice were utilized in the present study. These animals were killed at 72 hours after acute IS, and the brain infarct tissues were quickly harvested and prepared for the study.

Additionally, luzindole (5.0 μmol/L), an antagonist effect Mel, was utilized during cell culture for assessing the reversed cell protective effect of Mel.

Mel (M5250) and Luz (L2407) (ie antagonist of melatonin) were purchased from Sigma Chemical Co. Mel was dissolved in ethanol, and Luz was dissolved in DMSO immediately prior to utilization, respectively.

### 2.14 Statistical analysis

Statistical analysis was adequately performed by ANOVA followed by Bonferroni multiple comparison post hoc test. SAS statistical software for Windows version 8.2 (SAS institute) was utilized. A probability value <.05 is considered statistically significant.

## 3 RESULTS

### 3.1 In vitro pilot study assessed the expressions of TLRs dependently mediated the inflammatory downstream signalling in the presence and absence of Mel or IS^B6‐mouse^ brain tissue extract treatment in N2a cells (Figure 1)

**FIGURE 1:**
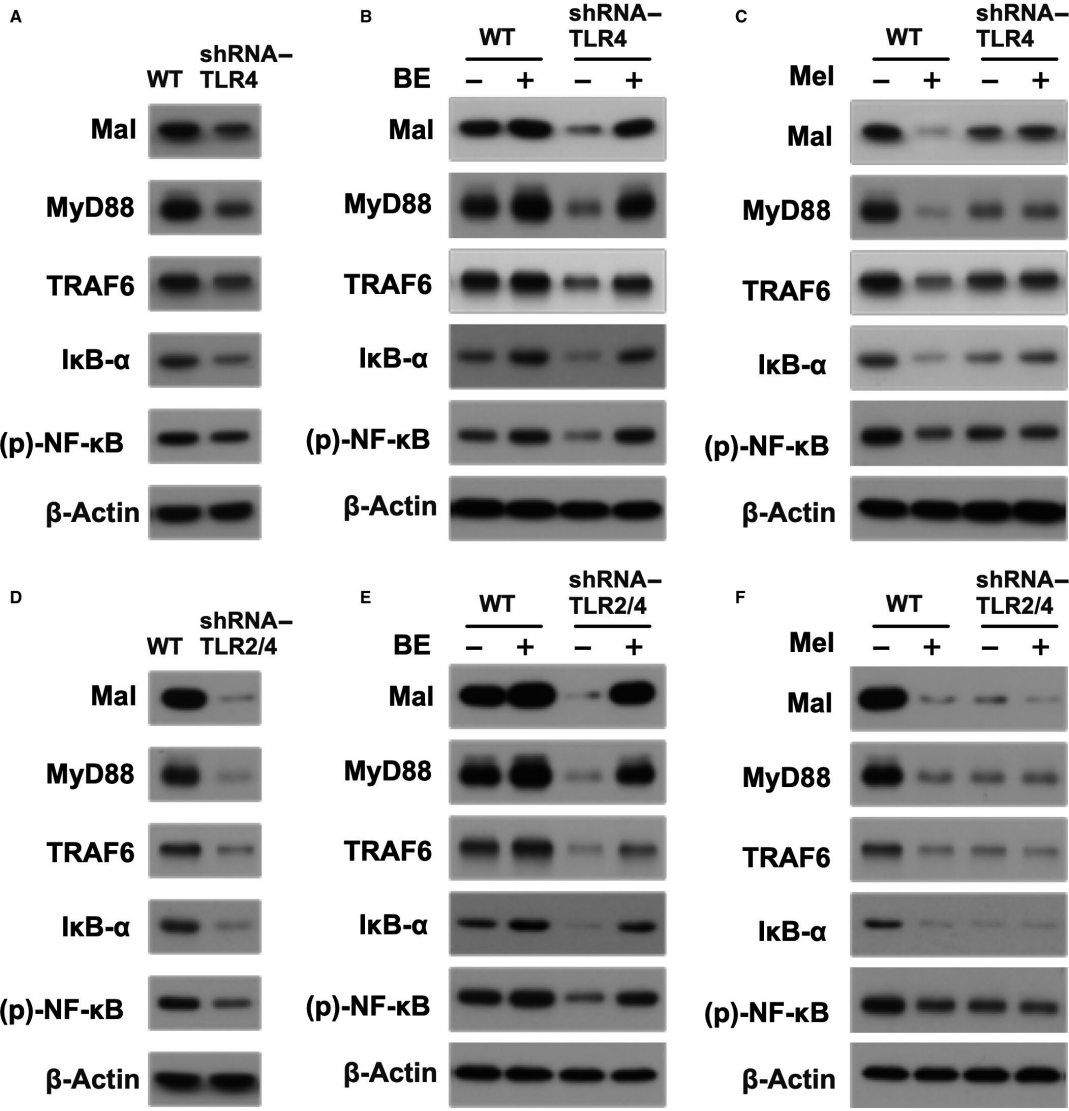
Pilot study for assessment of inflammatory downstream signalling in shRNA‐silencing TLR4 N2a cells and shRNA‐silencing TLR2/TLR4 (ie double silencing) N2a cells undergoing the BE or Mel treatment. Upper panel: Illustrating the protein expressions of MAL, MyD88, TRAF6, IκB‐α and phosphorylated (p)‐NF‐κB, that is the inflammatory signalling, in control and shRNA‐TLR4 N2a cells undergoing brain tissue extracts from ischaemic stroke B6 mouse (BE) and melatonin (Mel) treatment. A, As compared with the wild‐type (WT) (ie control group of N2a cells), the protein expressions of these parameters were notably suppressed in shRNA‐TLR4 N2a cells. B, On the other hand, the expressions of these parameters were notably increased in N2a cells undergoing the BE treatment that were partially reduced in shRNA‐TLR4 N2a cells undergoing BE treatment. C, As compared to the control group (ie WT), the protein expressions of these parameters were remarkably suppressed by Mel treatment. However, these parameters did not show notably change in shRNA‐TLR4 N2a cells undergoing the Mel treatment, implicating that Mel suppressed inflammatory signalling relying on the presence of TLR4. Lower panel: Illustrating the protein expressions of the inflammatory signalling, in control and shRNA‐TLR4 N2a cells and shRNA‐silencing TLR2/4 (ie TLR2 and TLR4 double knockdown) N2a cells undergoing the BE or Mel treatment. The results from (D), (E) and (F) demonstrated that as compared with the corresponding results from (A), (B) and (C), respectively, these protein expressions were further notably suppressed in shRNA‐silencing TLR2/4 N2a cells and lesser change by BE or Mel treatment in shRNA double silencing of TLR2 and TLR4 condition. MAL = MyD88 adaptor‐like; MyD88 = myeloid differentiation primary response 88; TRAF6 = tumour necrosis factor (TNF) receptor‐associated factor 6; TLR = Toll‐like receptor. IκB = nuclear factor of kappa light polypeptide gene enhancer in B‐cell inhibitor; NF = nuclear factor

As expected, the protein expressions of MAL, MyD88, TRAF6, IKB‐α and NF‐κB, five indices of inflammatory downstream signalling pathway, were markedly reduced in plasmid‐based shRNA TRL2/TLR4 (ie double knockdown resulted in TRL^2/4^‐silencing) N2a cells than in those of controls (ie culturing N2a cells without any treatment) (Figure 1: upper panel).

Additionally, these parameters were notably reduced in N2a cells treated by Mel as compared with the control group (ie vehicle). However, no additional alternation was encountered in TLR^2/4^‐silencing N2a cells with and without Mel treatment, suggesting that TLR2 and TLR4 play the critical roles for Mel to suppress this inflammatory downstream signalling axis (Figure 1: upper panel).

Furthermore, to elucidate whether DAMPs dependently interacted with TLR2 and TLR4 for activating this inflammatory downstream signalling axis, isolated IS^B6^‐B^EX^ (ie at a concentration of 100 µg/mL) were co‐cultured with N2a cells (ie vehicle) and TLR^2/4^‐silencing N2a cells (Figure 1: lower panel). The results showed that these protein expressions (ie Mal/MyD88/TRAF6/IKB‐α/NF‐κB) were markedly enhanced as compared with the controls that did not show further change in TLR^2/4^‐silencing N2a cells undergoing the Mel treatment, once again suggesting that Mel dependently mediated TLR2 and TLR4 to down‐regulate this inflammatory downstream signalling pathway (Figure 1: lower panel).

### 3.2 Na2 cell culture with IS^B6‐EX^ to mimic the preclinical setting of brain‐damaged released DAMPs for interacting with TLR2‐ and TLR4‐mediated inflammatory downstream signalling pathways that suppressed by Mel (Figures 2 and 3)

**FIGURE 2:**
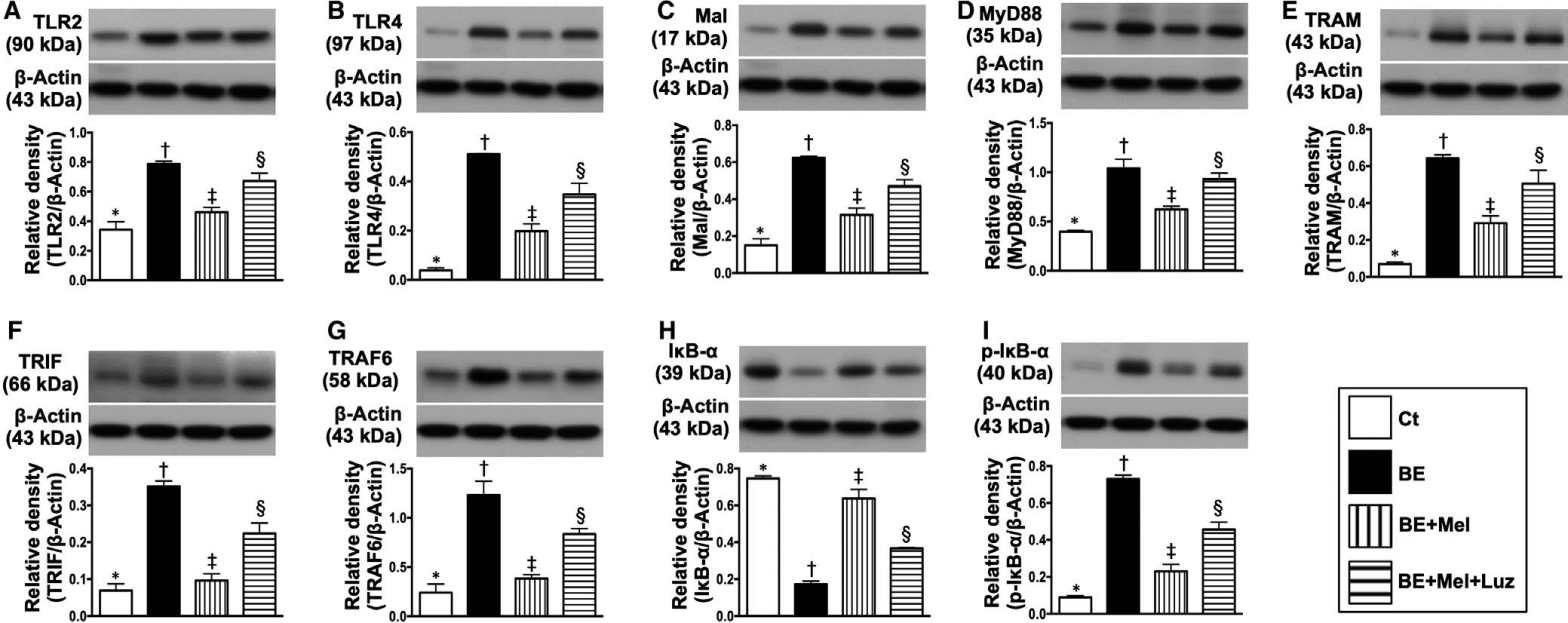
Melatonin suppressed DAMP‐dependent TLR2‐ and TLR4‐mediated inflammatory downstream signalling pathways in N2a cells. A, Protein expression of Toll‐like receptor 2 (TLR2), * vs other groups with different symbols (†, ‡, §), *P* < .001. B, Protein expression of TLR4, * vs other groups with different symbols (†, ‡, §), *P* < .001. C, Protein expression of MyD88 adaptor‐like (MAL), * vs other groups with different symbols (†, ‡, §), *P* < .001. D, Protein expression of myeloid differentiation primary response 88 (MyD88), * vs other groups with different symbols (†, ‡, §), *P* < .001. E, Protein expression of translocation‐associated membrane protein (TRAM), * vs other groups with different symbols (†, ‡, §), *P* < .001. F, Protein expression of TIR domain‐containing adapter‐inducing interferon‐β (TRIF), * vs other groups with different symbols (†, ‡, §), *P* < .0001. G, Protein expression of TNF receptor‐associated factor 6 (TRAF6), * vs other groups with different symbols (†, ‡, §), *P* < .001. H, Protein expression of nuclear factor of kappa light polypeptide gene enhancer in B‐cell inhibitor alpha (IκB‐α), * vs other groups with different symbols (†, ‡, §), *P* < .001. I, Phosphorylated (p)‐IκB‐α, * vs other groups with different symbols (†, ‡, §), *P* < .001. All statistical analyses were performed by one‐way ANOVA, followed by Bonferroni multiple comparison post hoc test (n = 4 for each group). Symbols (*, †, ‡, §) indicate significance (at 0.05 level). DAMPs = damage‐associated molecular patterns; BE = brain tissue extracts derived from ischaemic stroke B6 mouse; Ct = control; Mel = melatonin; Luz = luzindole

Based on the pilot study results of Figure 1, we utilized the cell culture (ie Na2 cell line) to further clarify the upstream and downstream signalling pathways of inflammatory reaction undergoing the IS^B6^‐B^EX^ treatment, that is mimicking the DAMPs released from brain‐damaged tissues to elicit inflammatory response. Additionally, Mel was utilized in this in vitro study to test its impact on inhibiting the inflammatory reaction. As we expected, the protein expressions of TLR2, TLR4, MAL/MyD88, TRAM/TRIF, TRAF6 and IκB‐α/p‐IκB‐α, a typical up‐ and downstream inflammatory signalling, were significantly increased in N2a + IS^B6^‐B^EX^ than in controls (ie N2a cells in vehicle) and were significantly reversed after Mel treatment (Figure 2). However, these parameters were significantly up‐regulated again after luzindole (ie an antagonist of Mel) treatment (Figure 2), highlighting that TLR2/TLR4 were essential for DAMPs to trigger the initiation and propagation of inflammation and Mel suppressed this downstream signalling of inflammation via an initial inhibition of TLR2/TLR4.

**FIGURE 3:**
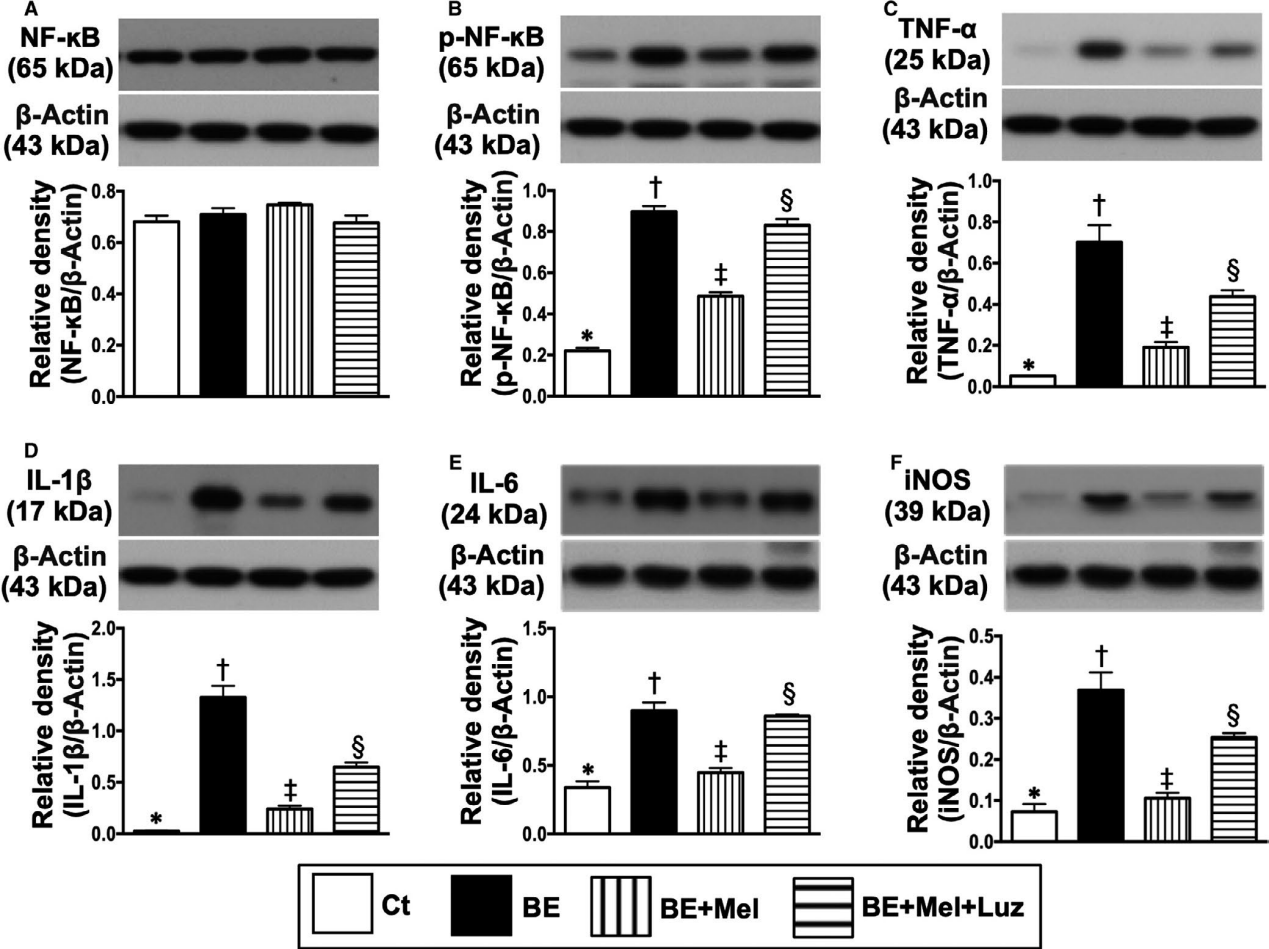
Melatonin suppressed downstream inflammatory biomarkers in N2a cells. A, Protein expression of total nuclear factor (NF)‐κB, *P* > .5. B, Protein expression of phosphorylated (p)‐NF‐κB, * vs other groups with different symbols (†, ‡, §), *P* < .001. C, Protein expression of tumour necrosis factor (TNF)‐α, * vs other groups with different symbols (†, ‡, §), *P* < .001. D, Protein expression of interleukin (IL)‐1β, * vs other groups with different symbols (†, ‡, §), *P* < .001. E, Protein expression of IL‐6, * vs other groups with different symbols (†, ‡, §), *P* < .001. F, Protein expression of induced nitric oxide synthase (iNOS), * vs other groups with different symbols (†, ‡, §), *P* < .0001. All statistical analyses were performed by one‐way ANOVA, followed by Bonferroni multiple comparison post hoc test (n = 4 for each group). Symbols (*, †, ‡, §) indicate significance (at 0.05 level). BE = brain tissue extracts derived from ischaemic stroke B6 mouse; Ct = control; Mel = melatonin; Luz = luzindole

The protein expressions of p‐NF‐κB, TNF‐α, IL‐1β, IL‐6 and iNOS, four translations of terminal downstream signalling inflammatory biomarkers, exhibited an identical pattern of TLR2/TLR4 among the groups (Figure 3).

### 3.3 Utilized the preclinical study to further elucidate the impacts of DAMP‐TLR2/TLR4 interaction and Mel on regulating the upstream and downstream signalling pathways (Figures 4‐7)

**FIGURE 4:**
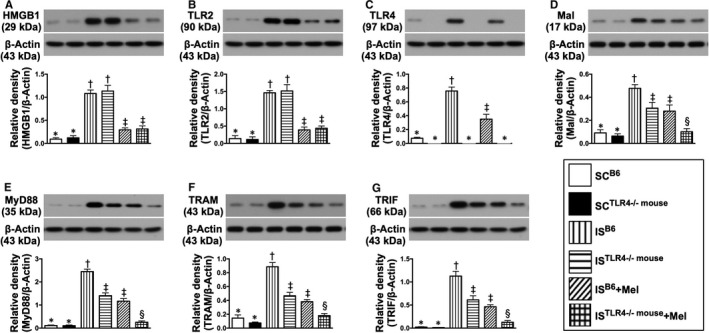
Melatonin therapy suppressed the upper part of inflammatory downstream signalling protein expressions in brain ischaemic area by day 28 after IS induction. A, Protein expression of high mobility group box 1 (HMGB1), * vs other groups with different symbols (†, ‡), *P* < .0001. B, Protein expression of Toll‐like receptor (TLR)2, * vs other groups with different symbols (†, ‡), *P* < .0001. C, Protein expression of TLR4, * vs other groups with different symbols (†, ‡), *P* < .0001. D, Protein expression of MyD88 adaptor‐like (MAL), * vs other groups with different symbols (†, ‡, §), *P* < .0001. E, Protein expression of myeloid differentiation primary response 88 (MyD88), * vs other groups with different symbols (†, ‡, §), *P* < .0001. F, Protein expression of translocation‐associated membrane protein (TRAM), * vs other groups with different symbols (†, ‡, §), *P* < .0001. G, Protein expression of TGF‐beta receptor‐interacting protein (TRIF), * vs other groups with different symbols (†, ‡, §), *P* < .0001. All statistical analyses were performed by one‐way ANOVA, followed by Bonferroni multiple comparison post hoc test (n = 6 for each group). Symbols (*, †, ‡, §) indicate significance (at 0.05 level). SC^B6^ = sham‐operated control in B6 mouse (ie wild‐type); SC^TLR4−/−mouse^ = sham‐operated control in TLR4 knockout (ie TLR4^−/−^) mouse; IS^B6^ = ischaemic stroke in B6 mouse; IS^TLR4−/−mouse^ = ischaemic stroke in TLR4 knockout mouse; Mel = melatonin

To assess the principal roles of DAMP‐TLR4 and Mel on regulating the inflammatory signalling pathways, the B6 and TLR4^−/−^ mice were utilized for the sham‐operated control (SC) and the acute IS model (ie preclinical study), respectively. By day 28 after acute IS induction, the results of brain specimen demonstrated that the protein expressions of HMGB1 (ie a component of DAMPs) and TLR2 were significantly lower in groups 1 (SC^B6 mouse^) and 2 (SC^TLR4−/− mouse^) than in groups 3 (IS^B6 mouse^), 4 (IS^TLR4−/− mouse^), 5 (IS^B6 mouse^ + Mel) and 6 (IS^TLR4−/− mouse^ + Mel) and significantly lower in groups 5 and 6 than in groups 3 and 4, but they showed no difference between groups 1 and 2, groups 3 and 4 or between groups 5 and 6 (Figure 4). These findings implicated that (a) the gene/protein expression of TLR2 was not attenuated in ^TLR4−/−^ mice, and (b) the protein expressions of HMGB1 and TLR2 were identically and remarkably suppressed by Mel in IS^TLR4−/− mouse^ as compared with IS^B6^ animals. On the other hand, the protein expression of TLR4 was not detected in groups 2, 4 and 6, but it showed significantly higher in group 3 than in groups 1 and 5, and significantly higher in group 5 than in group 1 (Figure 4).

**FIGURE 5:**
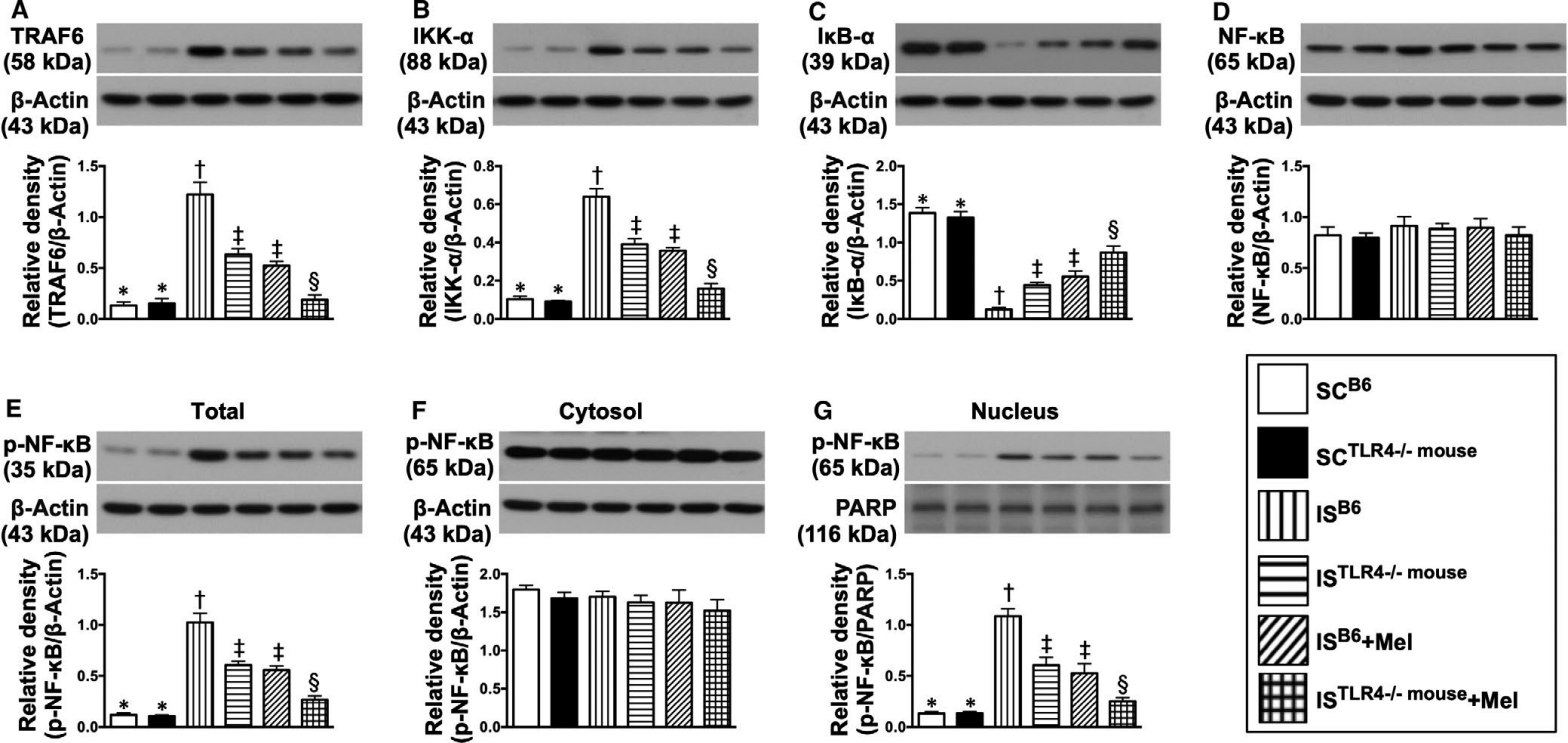
Melatonin therapy suppressed the middle part of inflammatory downstream signalling protein expressions in brain ischaemic area by day 28 after IS induction. A, Protein expression of tumour necrosis factor (TNF) receptor‐associated factor 6 (TRAF6), * vs other groups with different symbols (†, ‡, §), *P* < .0001. B, Protein expression of IKK‐α, * vs other groups with different symbols (†, ‡, §), *P* < .0001. C, Protein expression of IκB‐α, * vs other groups with different symbols (†, ‡, §), *P* < .0001. D, Protein expression of total NF‐κB, *P* > .5. E, Protein expression of total phosphorylated (p)‐NF‐κB, * vs other groups with different symbols (†, ‡, §), *P* < .0001. F, Protein expression of cytosolic NF‐κB, *P* > .5. G, Protein expression of nuclear‐NF‐κB (ie located in the nucleus), * vs other groups with different symbols (†, ‡, §), *P* < .0001. All statistical analyses were performed by one‐way ANOVA, followed by Bonferroni multiple comparison post hoc test (n = 6 for each group). Symbols (*, †, ‡, §) indicate significance (at 0.05 level). SC^B6^ = sham‐operated control in B6 mouse (ie wild‐type); SC^TLR4−/−mouse^ = sham‐operated control in TLR4 knockout (ie TLR4^−/−^) mouse; IS^B6^ = ischaemic stroke in B6 mouse; IS^TLR4−/−mouse^ = ischaemic stroke in TLR4 knockout mouse; Mel = melatonin

The protein expressions of Mal, MyD88, TRAM (Figure 4) and TRAF6 (Figure 5), four downstream mediators of TLR2/TLR4, were lowest in groups 1 and 2, highest in group 3, significantly higher in groups 4 and 5 than in group 6 and a non‐significantly statistical trend of being greater in group 4 than in group 5, but these parameters showed no difference between groups 1 and 2 (Figures 4 and 5). Interestingly, when the subgroup analysis was examined, we found that these parameters were remarkably reduced (ie about 50.0%) in group 4 (IS^TLR4−/− mouse^), further remarkably reduced (about 60.0%) in group 5 (IS^B6 mouse^ + Mel) and furthermore remarkably reduced (>85.0%) in group 6 (IS^TLR4−/−mouse^ + Mel) (Figures 4 and 5), suggesting that a net further reduction of these biomarkers in the same species (ie TLR4^−/−mouse^) was about 35.0% in group 6 as compared to group 4. Additionally, this finding (ie a net further reduction) may implicate that Mel treatment reduced the expressions of these parameters through not only TLR4 but also TLR2 and possible other signallings (although much less important) such as other TLRs.

Another interesting finding in the present study was that the protein expressions of these parameters did not differ between groups 4 (ie IS^TLR4−/−mouse^) and 5 (IS^B6 mouse^ + Mel), implying that the therapeutic effect of Mel on suppressing these inflammatory biomarkers was not inferior to the TLR4 genetic knockout condition (Figure 4).

TRAM and TRIF are specifically involved in the TLR4‐mediated TRAF6‐dependent signalling pathway. In the present study, we found that the protein expressions of these inflammatory mediators displayed an identical pattern of MyD88 (Figure 4).

The protein expressions of IKK‐α and IKB‐α are specifically involved in MyD88‐mediated NF‐κB‐dependent signalling. In the current study, we found that the protein expression of IKK‐α exhibited an identical pattern of MyD88 among six groups, whereas the protein expression of IKB‐α showed an opposite pattern of IKK‐α, suggesting an intrinsic consumption for active participation in the inflammatory signalling transmission (Figure 5). Additionally, the protein expression of p‐NF‐κB (ie located in cytosol) and nuclear‐NF‐κB (ie located in nucleus), two downstream signalling conductors of IKB‐γ/α/β, exhibited an identical pattern of MyD88 among the six groups (Figure 5).

**FIGURE 6:**
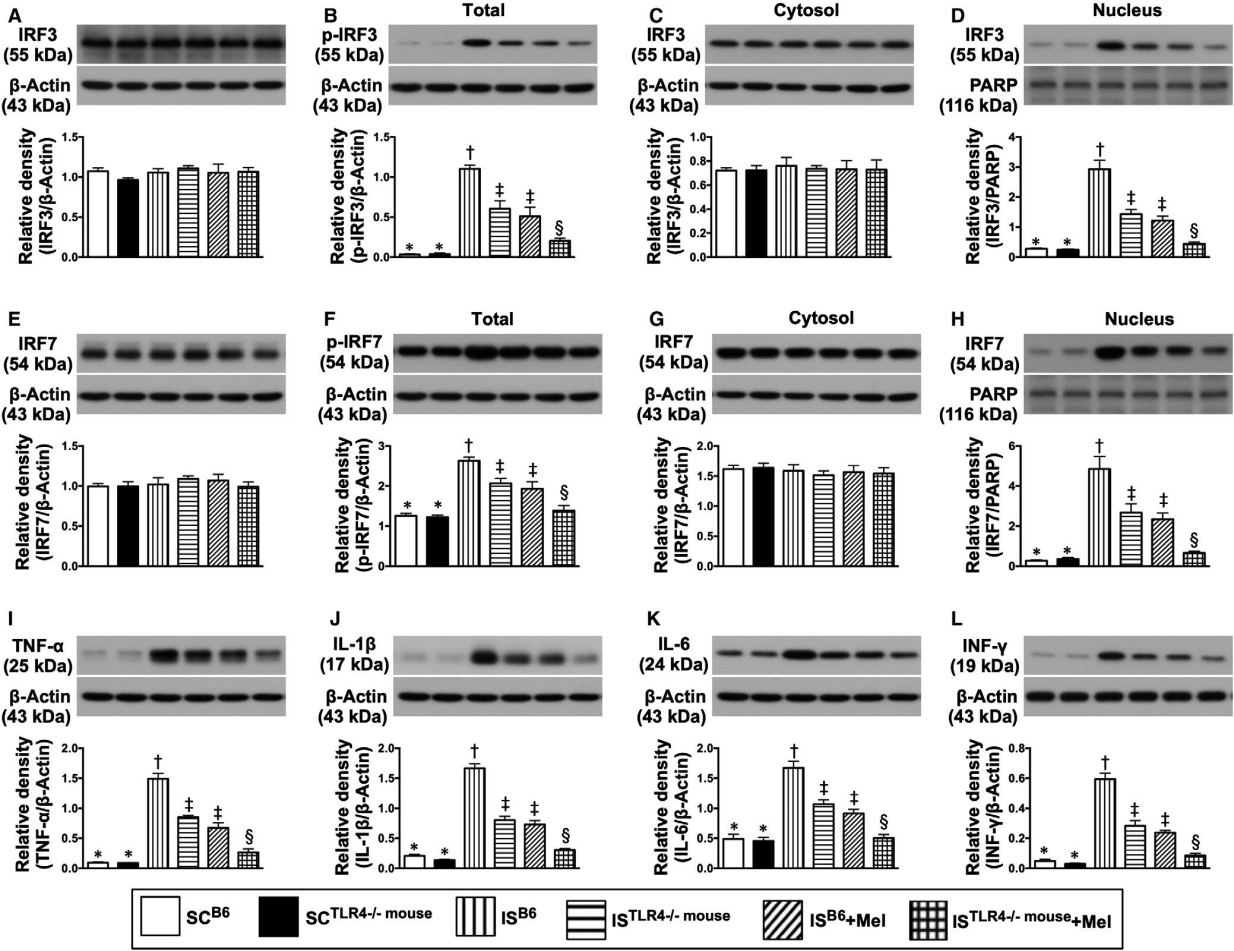
Melatonin therapy suppressed the terminal part of inflammatory downstream signalling protein expressions in brain ischaemic area by day 28 after IS induction. A, Protein expression of total interferon regulatory factor 1 (IRF3), *P* > .5. B, Protein expression of total phosphorylated (p)‐IRF3, * vs other groups with different symbols (†, ‡, §), *P* < .0001. C, Protein expression of cytosolic IRF3, *P* > .5. D, Protein expression of nuclear IRF3, * vs other groups with different symbols (†, ‡, §), *P* < .0001. E, Protein expression of total IRF7, *P* > .5. F, Protein expression of total p‐IRF7, * vs other groups with different symbols (†, ‡, §), *P* < .0001. G, Protein expression of cytosolic IRF7, *P* > .5. H, Protein expression of nuclear IRF7, * vs other groups with different symbols (†, ‡, §), *P* < .0001. I, Protein expression of tumour necrosis factor (TNF)‐α, * vs other groups with different symbols (†, ‡, §), *P* < .0001. J, Protein expression of interleukin (IL)‐1β, * vs other groups with different symbols (†, ‡, §), *P* < .0001. K, Protein expression of IL‐6, * vs other groups with different symbols (†, ‡, §), *P* < .0001. L, Protein expression of interferon (IFN)‐γ, * vs other groups with different symbols (†, ‡, §), *P* < .0001. All statistical analyses were performed by one‐way ANOVA, followed by Bonferroni multiple comparison post hoc test (n = 6 for each group). Symbols (*, †, ‡, §) indicate significance (at 0.05 level). SC^B6^ = sham‐operated control in B6 mouse (ie wild‐type); SC^TLR4−/−mouse^ = sham‐operated control in TLR4 knockout (ie TLR4^−/−^) mouse; IS^B6^ = ischaemic stroke in B6 mouse; IS^TLR4−/−mouse^ = ischaemic stroke in TLR4 knockout mouse; Mel = melatonin

The protein expressions of p‐IRF3/p‐IRF7 (ie located in cytosol) and nuclear‐IRF3/nuclear‐IRF7 (ie located in nuclei), four indices of terminal inflammatory signalling conductors, exhibited an identical pattern of MyD88 among the six groups (Figure 6).

Finally, the protein expressions of TNF‐α, IL‐1β, IL‐6 and IFN‐γ, four translation of cytokines (ie end‐products) by nuclei/DNA generation in response to the inflammatory signalling stimulations, exhibited an identical pattern of MyD88 among the six groups (Figure 6).

**FIGURE 7:**
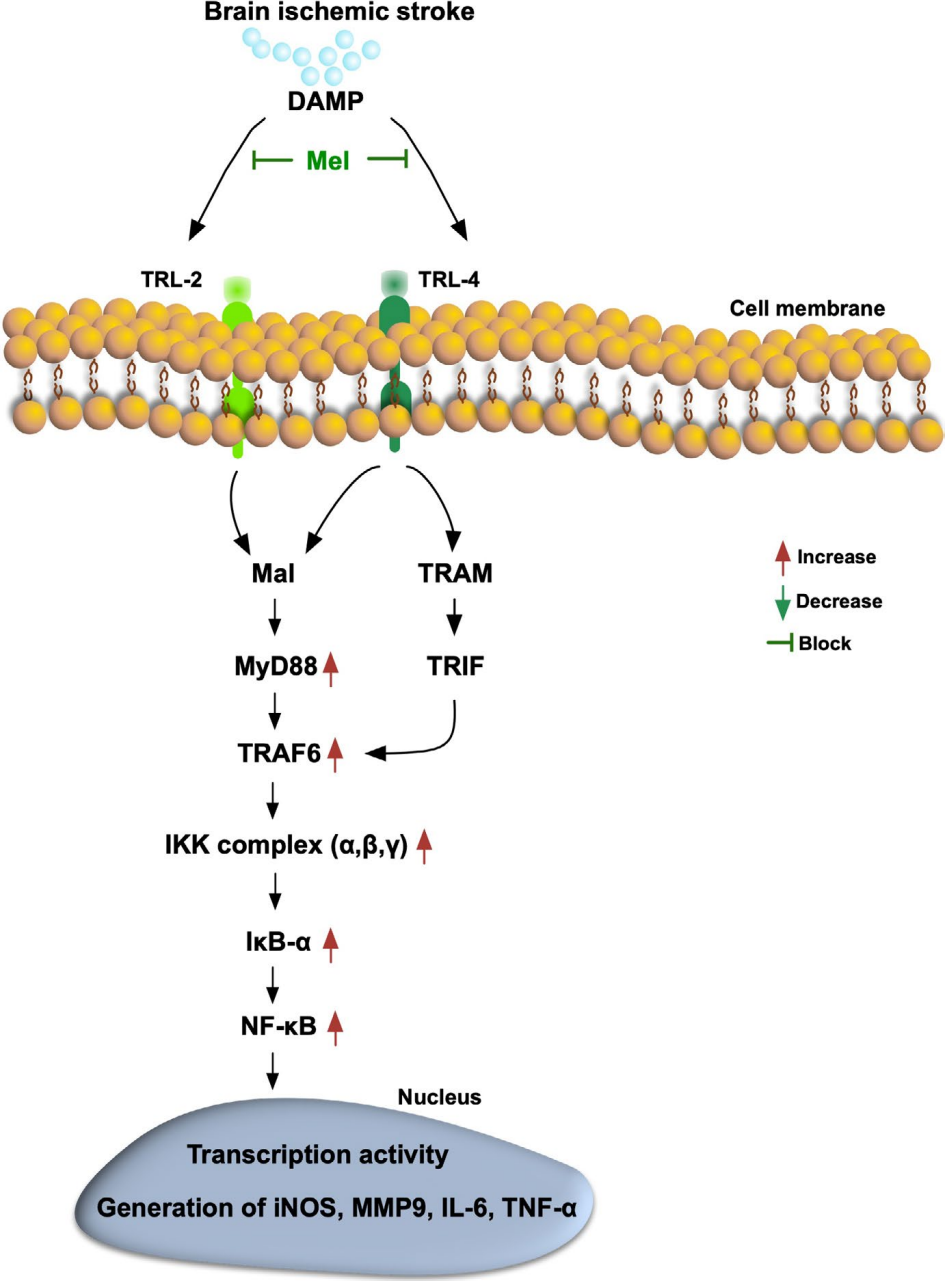
Based on the findings from Figures 1, 2, 3, 4, 5, 6, we summarized the proposed underlying mechanism of DAMP‐TLR inflammatory downstream signalling after acute IS. DAMP = damage‐associated molecular patterns; TLR = Toll‐like receptor; Mal = MyD88 adaptor‐like; TRAM = translocation‐associated membrane protein; TRIF = TGF‐beta receptor‐interacting protein; TRAF6 = TNF receptor‐associated factor; NF = nuclear factor; IL = interleukin; TNF = tumour necrosis factor; iNOS = inducible nitric oxide synthase

Accordingly, based on the findings from Figures 1, 2, 3, 4, 5, 6, we summarized the proposed underlying mechanism of DAMP‐TLR inflammatory downstream signalling pathway of acute IS that was suppressed by Mel treatment (Figure 7).

### 3.4 Protein expressions of oxidative stress signalling pathway, mitochondrial‐damaged biomarkers and cell death factors by day 28 after is induction (Figures 8 and 9)

**FIGURE 8:**
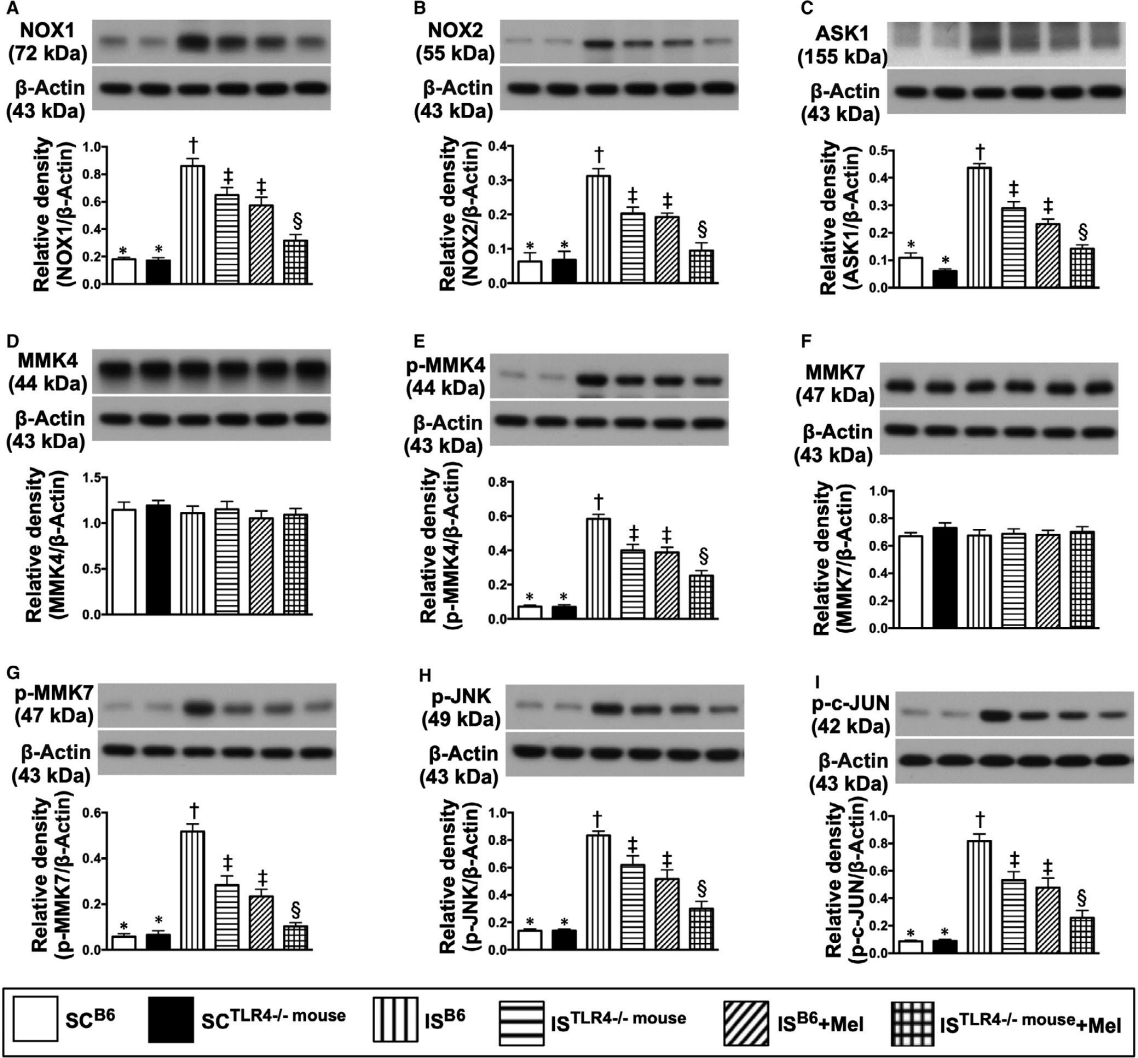
Protein expressions of oxidative stress downstream signalling pathway by day 28 after IS induction. A, Protein expression of NOX‐1, * vs other groups with different symbols (†, ‡, §), *P* < .0001. B, Protein expression of NOX‐2, * vs other groups with different symbols (†, ‡, §), *P* < .0001. C, Protein expression of apoptosis signal‐regulating kinase 1 (ASK1), * vs other groups with different symbols (†, ‡, §), *P* < .0001. D, Protein expression of total mitogen‐activated protein kinase kinase 4 (MMK4), *P* > .5. E, Protein expression of phosphorylated (p)‐MMK4, * vs other groups with different symbols (†, ‡, §), *P* < .0001. F, Protein expression of total MMK7, *P* > .5. G, Protein expression of p‐MMK7, * vs other groups with different symbols (†, ‡, §), *P* < .0001. H, Protein expression of phosphorylated c‐Jun N‐terminal kinase (p‐JNK), * vs other groups with different symbols (†, ‡, §), *P* < .0001. I, Protein expression of p‐c‐JUN, * vs other groups with different symbols (†, ‡, §), *P* < .0001. All statistical analyses were performed by one‐way ANOVA, followed by Bonferroni multiple comparison post hoc test (n = 6 for each group). Symbols (*, †, ‡, §) indicate significance (at 0.05 level). SC^B6^ = sham‐operated control in B6 mouse (ie wild‐type); SC^TLR4−/−mouse^ = sham‐operated control in TLR4 knockout (ie TLR4^−/−^) mouse; IS^B6^ = ischaemic stroke in B6 mouse; IS^TLR4−/−mouse^ = ischaemic stroke in TLR4 knockout mouse; Mel = melatonin

The protein expressions of NOX‐1 and NOX‐2, two indicators of oxidative stress, were lowest in groups 1 and 2, highest in group 3, significantly higher in groups 4 and 5 than in group 6, but they showed no significant difference between groups 1 and 2 or between groups 4 and 5 (Figure 8). Additionally, apoptosis signal‐regulating kinase 1 (ASK1), an activator of c‐JNK and p38 mitogen‐activated protein kinases in a Raf‐independent fashion in response to oxidative stress, displayed an identical pattern of oxidative stress protein among the six groups (Figure 8). Furthermore, the protein expressions of p‐MKK4, p‐MKK7, p‐JNK and p‐c‐JUN, four downstream signalling mediators of oxidative stress, exhibited an identical pattern of NOX‐1/NOX‐2 among the six groups (Figure 8).

**FIGURE 9:**
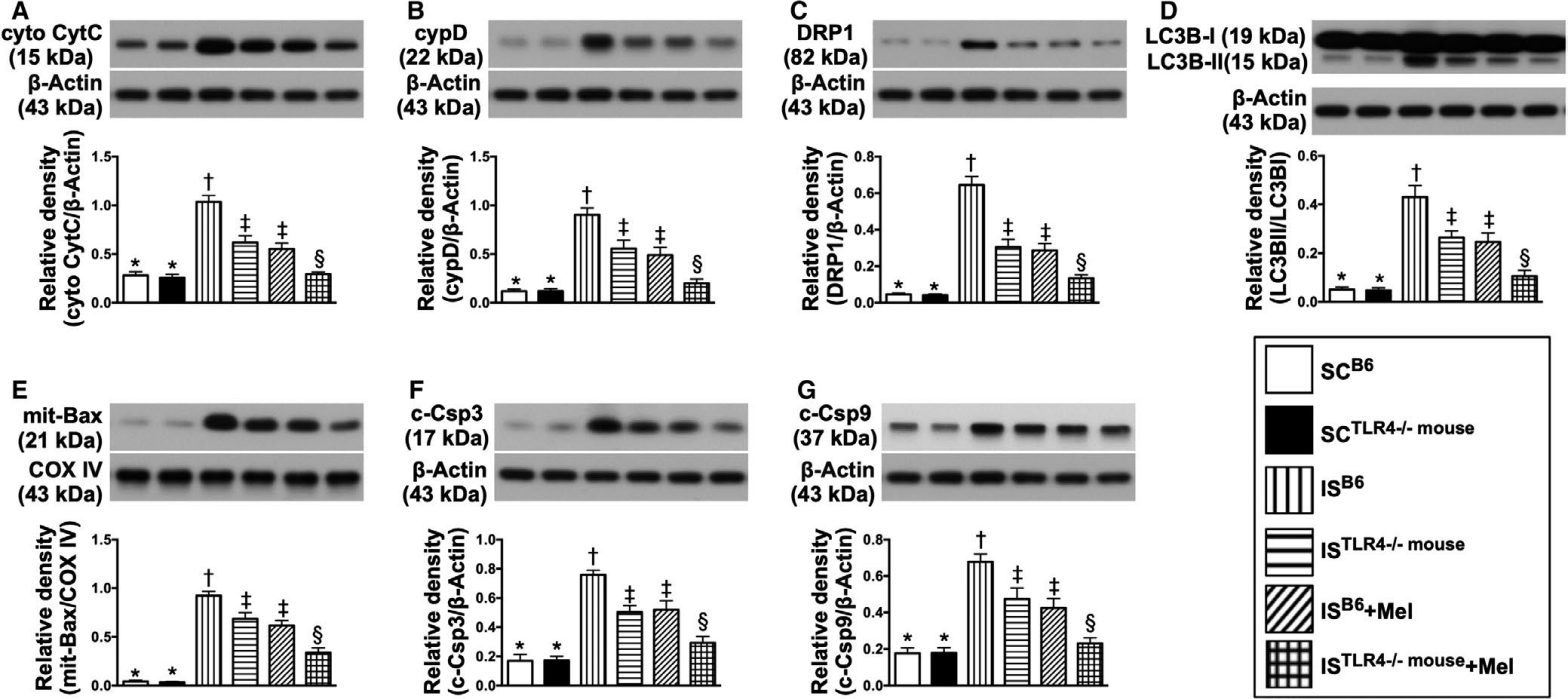
Mitochondrial‐damaged biomarkers and cell death factors by day 28 after IS induction. A, Protein expression of cytosolic cytochrome C (cyto‐CytC), * vs other groups with different symbols (†, ‡, §), *P* < .0001. B, Protein expression of cyclophilin D (cypD), * vs other groups with different symbols (†, ‡, §), *P* < .0001. C, Protein expression of dynamin‐related protein 1 (DRP1), * vs other groups with different symbols (†, ‡, §), *P* < .0001. D, The ratio of protein expression of LC3B‐II to LC3B‐I, * vs other groups with different symbols (†, ‡, §), *P* < .0001. E, Protein expression of mitochondrial (Mit)‐Bax, * vs other groups with different symbols (†, ‡, §), *P* < .0001. F, Protein expression of cleaved caspase 3 (c‐Csp3), * vs other groups with different symbols (†, ‡, §), *P* < .0001. G, Protein expression of cleaved caspase 9 (c‐Csp9), * vs other groups with different symbols (†, ‡, §), *P* < .0001. All statistical analyses were performed by one‐way ANOVA, followed by Bonferroni multiple comparison post hoc test (n = 6 for each group). Symbols (*, †, ‡, §) indicate significance (at 0.05 level). SC^B6^ = sham‐operated control in B6 mouse (ie wild‐type); SC^TLR4−/−mouse^ = sham‐operated control in TLR4 knockout (ie TLR4^−/−^) mouse; IS^B6^ = ischaemic stroke in B6 mouse; IS^TLR4−/−mouse^ = ischaemic stroke in TLR4 knockout mouse; Mel = melatonin

The protein expressions of cytosolic cytochrome C, cyclophilin D, DRP1, three indicators of mitochondrial damage biomarkers and ratio of LC3B‐II to LC3B‐I, an indicator of autophagy, displayed a similar pattern to those of oxidative stress biomarkers among the six groups (Figure 9).

The protein expressions of mitochondrial Bax, caspase 3 and 9, three indicators of cell death factors, revealed an identical pattern of oxidative stress biomarkers among the six groups (Figure 9).

**FIGURE 10:**
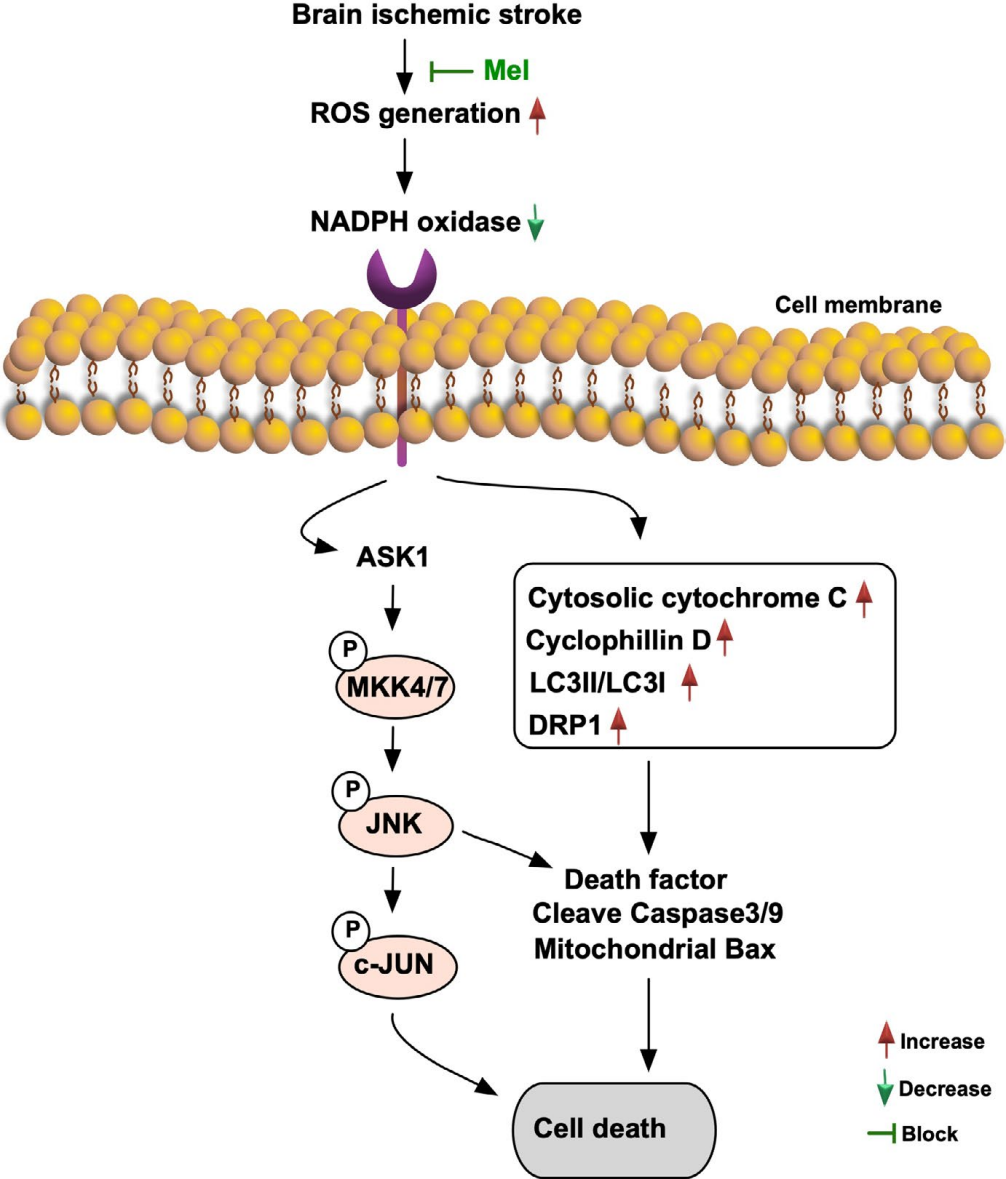
Based on the findings from Figures 8 and 9, we summarized the proposed underlying mechanism of oxidative stress downstream signalling pathway participated in brain damage in setting of acute IS. Mel = melatonin; ROS = reactive oxygen species (ROS); ASK1 = apoptosis signal‐regulating kinase 1; MMK = mitogen‐activated protein kinase kinase; DRP1 = dynamin‐related protein 1

Accordingly, based on the findings from Figures 8 and 9, we summarized the proposed underlying mechanism of oxidative stress downstream signalling pathway involved in brain damage in setting of acute IS (Figure 10).

### 3.5 BIV by day 28 after acute is induction (Figure 11)

**FIGURE 11:**
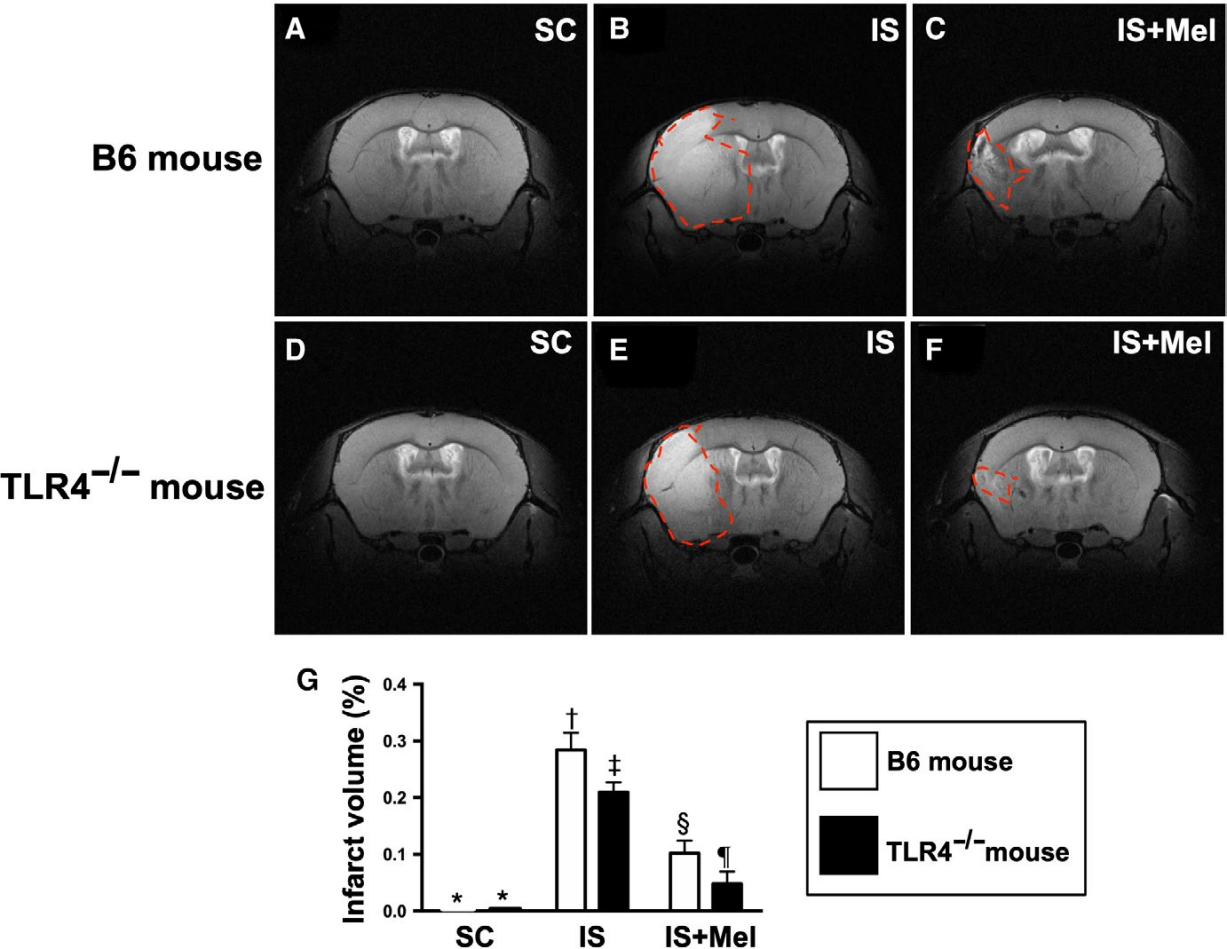
Brian MRI determined the brain infarct volume by day 28 after acute IS induction. A‐F, Illustrating the brain magnetic resonance imaging (MRI) finding for identification of brain infarct zone (white colour) (red dotted line area). G, Analytical result of brain infarct volume, * vs other groups with different symbols (†, ‡, §, ¶), *P* < .0001. All statistical analyses were performed by one‐way ANOVA, followed by Bonferroni multiple comparison post hoc test (n = 4 for each group). Symbols (*, †, ‡, §, ¶) indicate significance (at 0.05 level). SC = sham‐operated control; IS = ischaemic stroke; Mel = melatonin; TLR4^−/−^ = TLR4 knockout

The brain MRI demonstrated that the BIV was lowest in groups 1 and 2, highest in group 3, significantly higher in group 4 than in groups 5 and 6 and significantly higher in group 5 than group 6 but it showed no significant difference between groups 1 and 2 (Figure 11).

### 3.6 Time courses of neurological function examinations and the BIA (Figure 12)

**FIGURE 12:**
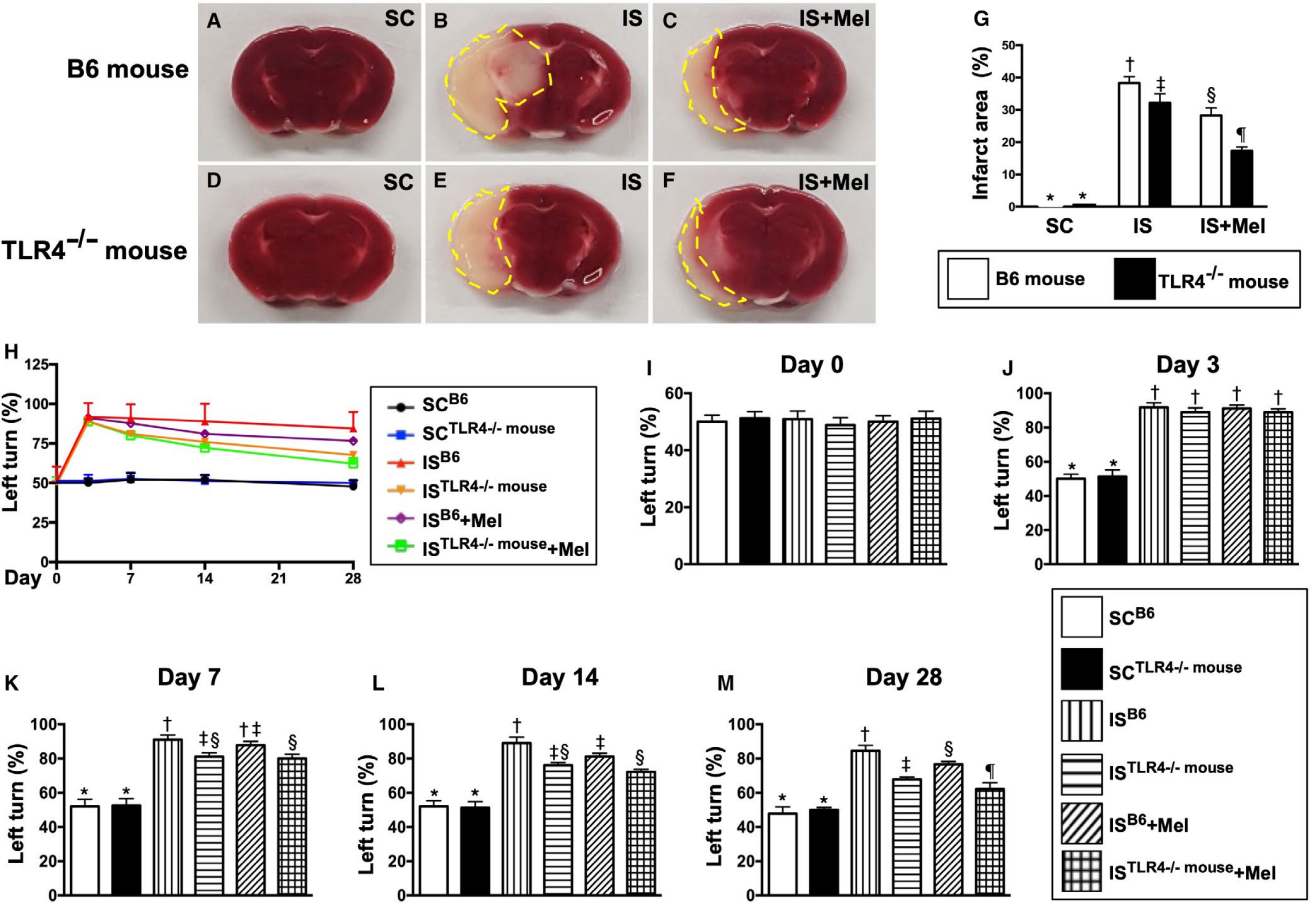
Pathological findings of brain infarct area by day 14 and time courses of neurological function after acute IS. A‐F, Illustrating the triphenyltetrazolium chloride (TTC) (100×) of whole brain cross section for identification of brain infarct area (BIA) (the yellow dotted line indicated the boundary of BIA) by day 14 after IS procedure (n = 4). G, Statistical analysis of summated (five brain cross sections in each animal) BIA, * vs other groups with different symbols (†, ‡, §, ¶), *P* < .0001. H, Illustrating the corner test for determining neurological function among days 0, 3, 7, 14 and 28 after acute IS procedure. I, Statistical analysis of neurological function (ie by corner test) by day 0, *P* > .5. J, Statistical analysis of neurological function day 3, * vs †, *P* < .001. K, Statistical analysis by day 7, * vs other groups with different symbols (†, ‡, §), *P* < .0001. L) Statistical analysis by day 14, * vs other groups with different symbols (†, ‡, §), *P* < .001. M, Statistical analysis by day 28, * vs other groups with different symbols (†, ‡, §, ¶), *P* < .0001. All statistical analyses were performed by one‐way ANOVA, followed by Bonferroni multiple comparison post hoc test (n = 6 for each group). Symbols (*, †, ‡, §, ¶) indicate significance (at 0.05 level). SC^B6^ = sham‐operated control in B6 mouse (ie wild‐type); SC^TLR4−/−mouse^ = sham‐operated control in TLR4 knockout (ie TLR4^−/−^) mouse; IS^B6^ = ischaemic stroke in B6 mouse; IS^TLR4−/−mouse^ = ischaemic stroke in TLR4 knockout mouse; Mel = melatonin

By day 14 after IS induction, the TTC stain demonstrated that the BIA was lowest in groups 1 and 2, highest in group 3, significantly higher in group 4 than in groups 5 and 6 and significantly higher in group 5 than in group 6 (Figure 12).

By day 0, the neurological function (ie by corner test) did not differ among the six groups. However, by day 3, the neurological function was significantly impaired in groups 3 to 6 than in groups 1 and 3, but it showed no different among the former four groups or between the latter two groups. Furthermore, by days 7, 14 and 28, the neurological function was significantly impaired in group 3 than in groups 1 and 2, while significantly progressively improved in group 5 and further improved in groups 4 and 6.

### 3.7 The 3rd day's circulating levels of inflammatory cells and inflammatory cell infiltrations in BIA by day 28 after acute is induction (Figure 13)

**FIGURE 13:**
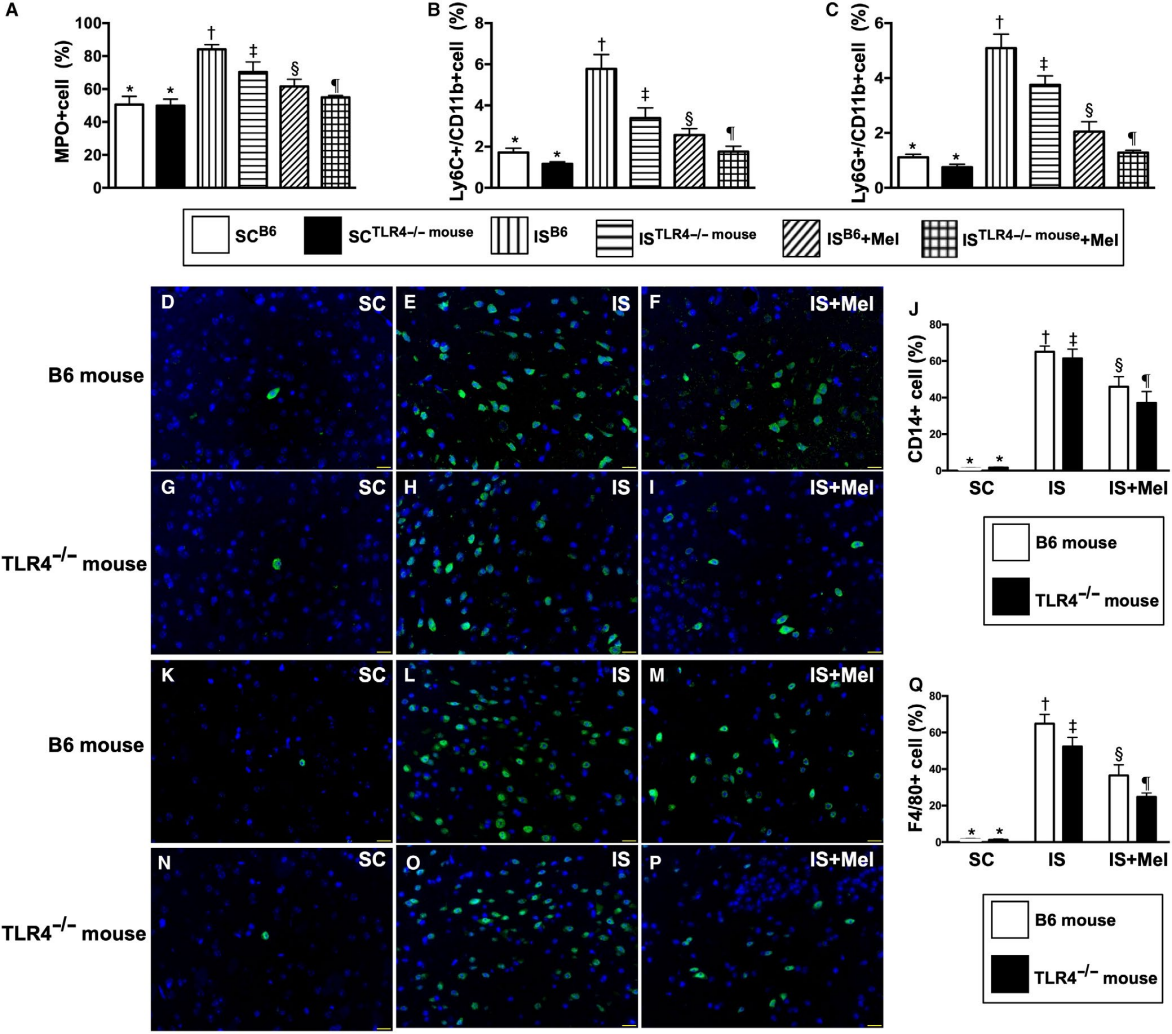
Circulating number of inflammatory cells at 3rd day and inflammatory cell expressions in brain infarct area (BIA) by day 28 after acute IS induction. A, Circulating number of myeloperoxidase (MPO)+ cells, * vs other groups with different symbols (†, ‡, §, ¶), *P* < .0001. B, Circulating number of Ly6C+/CD11b + cells, * vs other groups with different symbols (†, ‡, §, ¶), *P* < .0001. C, Circulating number of Ly6G+/CD11b + cells, * vs other groups with different symbols (†, ‡, §, ¶), *P* < .0001. D‐I, Immunofluorescent (IF) microscopic finding (400×) for identification of cellular expression of CD14 in BIA (green colour). J, Analytical result of number of CD14 + cells, * vs other groups with different symbols (†, ‡, §, ¶), *P* < .0001. K‐P, IF microscopic finding (400×) for identification of cellular expression of F4/80 in BIA (green colour). Q, Analytical result of number of F4+/80 cells, * vs other groups with different symbols (†, ‡, §, ¶), *P* < .0001. All statistical analyses were performed by one‐way ANOVA, followed by Bonferroni multiple comparison post hoc test (n = 6 for each group). Symbols (*, †, ‡, §, ¶) indicate significance (at 0.05 level). SC^B6^ = sham‐operated control in B6 mouse (ie wild‐type); SC^TLR4−/−mouse^ = sham‐operated control in TLR4 knockout (ie TLR4^−/−^) mouse; IS^B6^ = ischaemic stroke in B6 mouse; IS^TLR4−/−mouse^ = ischaemic stroke in TLR4 knockout mouse; Mel = melatonin

By day 3 after acute IS procedure, the circulating numbers of myeloperoxidase (MPO)+, Ly6C+/CD11b+ and Ly6G+/CD11b+ cells, three indices of inflammation, were lowest in groups 1 and 2, highest in group 3, significantly higher in group 4 than in groups 5 and 6 and significantly higher in group 5 than in group 6. Additionally, by day 28 after IS procedure, the cellular expressions of CD14 and F4/80 in BIA, another two indicators of inflammation, displayed an identical pattern to the circulating inflammatory cells among the six groups (Figure 13).

## 4 DISCUSSION

This study utilizing in vitro, in vivo and TLR4‐/‐ mouse IS model studies to investigate the Mel and TLR4 on regulating the DAMP‐TLR‐MyD88‐TRIF‐TRAF6 inflammatory and oxidative stress signalling pathways, yielded several striking implications. First, acute IS not only elicited a complex inflammatory signalling but also initiated the oxidative stress signalling which in turn further damaged the molecular‐cellular levels of brain architecture and neurological function (refer to Figures 7 and 10). Second, the current study demonstrated that DAMPs not only interacted with TLR4 but also co‐ordinated with TLR2 (or other TLRs) to commence inflammatory downstream signalling pathways. Third, Mel was recognized to own pleiotropic effects on protecting the brain organ from IS injury via inhibiting anti‐inflammation and antioxidative stress.

One important finding of in vitro study was that ischaemic brain tissue extracts (ie DAMPs from IS^B6^‐B^EX^) played a crucial role on commencing MyD88‐TRIF‐TRAF6‐NF‐κB/IRF inflammatory signalling. Another important finding was that the in vitro study further discovered that the TLR4 played a dominant role on transmitting this downstream inflammatory signalling in N2a cells. Intriguingly, by using a brain death (BD) animal model, we have previously also found that DAMPs derived from BD^EX^ were crucial for spreading the downstream inflammatory signalling of TLR4‐MyD88/Iκ‐B/NF‐κB.^10^ In this way, our present findings were consistent with those of our previous study.^10^ Importantly, by using shRNA‐silencing TLR2/4 in N2a cells, we identified that activation and transmission of inflammatory downstream signalling were TLR2/TLR4‐dependent, highlighting that interaction between DAMPs and TLR2/4 was the most important checkpoint for inflammatory axis. Of distinctive importance was that Mel treatment acted similarly to TLR2/4‐silencing effect on down‐regulating the inflammatory signalling. Accordingly, our results extended the finding from our previous study.^10^

A principal finding in the present study was that time‐points of corner test established that the neurological function was markedly improved in groups 4 and 5 and more remarkably improved in group 6 than in group 3. Interestingly, previous preclinical studies have also showed that Mel treatment significantly preserved the integrity of neurological function in ischaemia/ischaemia‐reperfusion injury.^31^, ^32^

The most novel finding in the present study was that the brain MRI (ie imaging study) TTC stain (ie pathological finding) demonstrated that the BIV was substantially reduced in groups 4/5 and more substantially reduced in group 6 as compared with group 2. These findings could explain why the neurological outcome was much better in groups 4 to 6 than in that of group 3.

It was noteworthy that as compared with group 3 animals, a great reduction in both BIA and BIV was found in group 4 animals, highlighting that TLR4 played a critical role on participating the brain organ damage in setting of IS. Another noteworthy point was that as compared with group 4, wide margins of reduction in BIA and BIV were found in group 5, highlighting that Mel was not inferior to the role of deleting TRL4 on protecting brain integrity against IS‐induced damage. Of most distinctive issue was that the BIA and BIV were much lower in group 6 than in group 5 (ie ratio of brain infarct volume reduction in IS^TLR4−/−mouse^ + Mel vs IS^B6^ mouse + Mel >1.0‐fold) implicating that not only TLR4 (despite its role was proved to be dominant by the current study) but also TLR2 (ie proved by our previous study)^10^ as well as other possible contributors combined to involve in the destruction of brain architecture and deterioration of neurological function after acute IS.

The associations between generation of oxidative stress and mitochondrial damage and cell apoptosis and organ damage in setting of ischaemia have been extensively addressed by previous studies.^6^, ^7^, ^10^, ^15^, ^17^, ^18^, ^19^, ^28^ An essential finding in the present study was that the oxidative and mitochondrial‐damaged markers were notably increased in IS animals. Additionally, the oxidative stress downstream signalling pathway (refer to Figure 5) was clearly delineated in these IS animals (ie group 3). Importantly, all of these parameters were notably reduced in groups 4/5 and further notably reduced in group 6 animals. Accordingly, our findings, being consistent with the findings of previous studies,^6^, ^7^, ^10^, ^15^, ^17^, ^18^, ^19^, ^28^ not only could explain why the brain damage was notably lesser and the neurological function was more preserved in groups 4 to 6 animals but also could explain our hypothesis that “other contributors gathered together” to involve in the brain destruction and neurological dysfunction after acute IS.

It is well recognized that inflammation aggravates brain damage after acute IS,^1^, ^2^, ^3^, ^4^, ^5^, ^6^, ^7^ causing irreversibly neurological sequelae.^1^, ^2^, ^3^, ^4^, ^5^, ^6^, ^7^ A fundamental finding in the present study was that the cellular‐molecular levels (ie in tissue and circulation) of inflammatory mediators were remarkably increased in group 3 animals as compared with groups 1/2, highlighting that these terminal signallings of inflammatory mediators were the results of DAMP‐TLR interaction after acute IS. Our findings, in addition to reinforcing the findings of previous studies,^1^, ^2^, ^3^, ^4^, ^5^, ^6^, ^7^ could, at least in part, explain why the brain damage was remarkably present in these IS animals. Conformingly, the cellular‐molecular perturbations and brain damage were substantially reduced, whereas the neurological function was notably preserved in groups 4/5 and further improved in the group 6.

## 5 STUDY LIMITATION

This study has limitations. First, the study period was 28 days. Accordingly, the long‐term effect of Mel therapy on protecting brain from IS damage is currently unclear. Additionally, the pathophysiological changes were completed at 28 day after ischaemia. Theoretically, short‐term studies (acute phase) are more valuable for signalling analyses, especially when innate inflammatory immune‐related downstream signalling was taken into investigations. However, this study did not provide this information. Second, the limitation for the clinical translation of the treatment with melatonin was that the Mel treatment began 3 days prior to the IS. Third, the behavioural experiments did not investigate in the present study that would, therefore, affect the precise and comprehensive estimation of neurological recovery after Mel treatment.

In conclusion, Mel therapy effectively preserved the integrities of brain architecture and neurological function in setting of acute IS in mice mainly through regulating the inflammatory and oxidative stress signalling pathways.

## CONFLICT OF INTEREST

The authors declare that there is no conflict of interest associated with this manuscript.

## AUTHOR CONTRIBUTION

**Kuan‐Hung Chen:** Conceptualization (equal); Project administration (equal); Supervision (equal); Writing‐original draft (equal); Writing‐review & editing (equal). **Kun‐Chen Lin:** Data curation (equal); Visualization (equal). **Sheung‐Fat Ko:** Methodology (equal); Validation (equal); Visualization (equal). **John Y. Chiang:** Formal analysis (equal); Methodology (equal); Validation (equal); Visualization (equal). **Jun Guo:** Conceptualization (equal); Supervision (equal); Writing‐original draft (equal); Writing‐review & editing (equal). **Hon‐Kan Yip:** Conceptualization (equal); Funding acquisition (equal); Project administration (equal); Supervision (equal); Writing‐original draft (equal); Writing‐review & editing (equal).

## Data Availability

The data sets of present study can be available from the corresponding author upon request (han.gung@msa.hinet.net).
